# Patient-Specific Retinal Organoids Recapitulate Disease Features of Late-Onset Retinitis Pigmentosa

**DOI:** 10.3389/fcell.2020.00128

**Published:** 2020-03-06

**Authors:** Mei-Ling Gao, Xin-Lan Lei, Fang Han, Kai-Wen He, Si-Qian Jin, You-You Zhang, Zi-Bing Jin

**Affiliations:** ^1^Laboratory of Stem Cell & Retinal Regeneration, Institute of Stem Cell Research, Division of Ophthalmic Genetics, The Eye Hospital, Wenzhou Medical University, Wenzhou, China; ^2^National Center for International Research in Regenerative Medicine and Neurogenetics, National Clinical Research Center for Ophthalmology, State Key Laboratory of Ophthalmology, Optometry and Visual Science, Wenzhou, China

**Keywords:** late onset, retinitis pigmentosa, iPSCs, PDE6B, retinal organoids

## Abstract

Although an increasing number of disease genes have been identified, the exact cellular mechanisms of retinitis pigmentosa (RP) remain largely unclear. Retinal organoids (ROs) derived from the induced pluripotent stem cells (iPSCs) of patients provide a potential but unvalidated platform for deciphering disease mechanisms and an advantageous tool for preclinical testing of new treatments. Notably, early-onset RP has been extensively recapitulated by patient-iPSC-derived ROs. However, it remains a challenge to model late-onset disease in a dish due to its chronicity, complexity, and instability. Here, we generated ROs from late-onset RP proband-derived iPSCs harboring a *PDE6B* mutation. Transcriptome analysis revealed a remarkably distinct gene expression profile in the patient ROs at differentiation day (D) 230. Changes in the expression genes regulating cGMP hydrolysis prompted the elevation of cGMP levels, which was verified by a cGMP enzyme-linked immunosorbent assay (ELISA) in patient ROs. Furthermore, significantly higher cGMP levels in patient ROs than in control ROs at D193 and D230 might lead to impaired formation of synaptic connections and the connecting cilium in photoreceptor cells. In this study, we established the first late-onset RP model with a consistent phenotype using an *in vitro* cell culture system and provided new insights into the PDE6B-related mechanism of RP.

## Introduction

Retinitis pigmentosa (RP) has a prevalence of approximately one in 4000, affecting approximately 1.5 million individuals in total worldwide (Hartong et al., 2006). RP, a hereditary retinal degenerative disease, is characterized by irreversible loss of photoreceptor cells. At the cellular level, this syndrome correlates with the primary degeneration of rod photoreceptors. In the progressive stage of this disease, the cone photoreceptor system might be affected, which eventually causes daytime vision impairment or complete visual loss (Hartong et al., 2006). Mutations in the gene encoding the beta subunit of rod cGMP-phosphodiesterase type 6 (*PDE6B*) account for 4 to 5% of autosomal recessive RP (Danciger et al., 1995). Recently, *PDE6B* mutation was identified as disease-causing gene in 2.4% patients from a large cohort of 1095 patients with RP (Khateb et al., 2019). Prevalent mouse models harboring *Pde6b* mutations, *rd1*, and *rd10* have been widely used for deciphering pathogenesis and examining novel therapeutics for RP (Bowes et al., 1990; Barhoum et al., 2008).

Progressive loss of rod and cone photoreceptors accompanied by elevated concentration of cGMP and an influx of Ca^2+^ through the CNG channel can be observed in animal RP models, which resemble the phenotype seen in human disease. However, interspecies variation, such as differences in retinal structure and life rhythm, limits the use of animal models in mechanistic studies and especially in preclinical studies. Conflicting results have been observed in mice and canines (Frasson et al., 1999; Pearce-Kelling et al., 2001) when used for drug testing (calcium channel blockers). Recently, patient-specific induced pluripotent stem cells (iPSCs) combined with differentiation technology have provided an unlimited cell source for disease modeling and can mimic primary disease tissue after appropriate induction (Rossi et al., 2018; Ballard et al., 2019; Foltz and Clegg, 2019). Retinal organoids (ROs) have been successfully generated from human embryonic stem cells (ESCs) and iPSCs (Llonch et al., 2018; Jin et al., 2019). These ROs, generated in dishes, display proper neural retina markers (DiStefano et al., 2018), form retinal stratification with apical-basal polarity (Hasegawa et al., 2016), and even possess light responses (Zhong et al., 2014; Hallam et al., 2018). Furthermore, retinogenesis can be recapitulated in ROs derived from pluripotent stem cells and analyzed by transcriptomic analysis (Kaewkhaw et al., 2015; Voelkner et al., 2016), and single-cell RNA-seq (Collin et al., 2019; Kim et al., 2019; Mao et al., 2019). Thus, patient-specific iPSCs combined with a three-dimensional (3D) culture system can generate unlimited cell sources for personalized drug testing and organ replacement (Shimada et al., 2017; Buskin et al., 2018; Deng et al., 2018; Guo et al., 2019; Li et al., 2019).

Disease models with ROs for early-onset retinal degeneration have been established by us and others (Shimada et al., 2017; Deng et al., 2018). Defects in retinal development and ciliopathy in photoreceptors have been found in *RPGR* and *CEP290* mutant ROs. In the present study, we established a patient-iPSC-derived RO model harboring a *PDE6B* mutation. Transcriptomic analysis and morphology demonstrate relatively normal retinal development of PDE6B patient ROs compared with control ROs before differentiation day (D) 180. At D230, however, mislocated rod photoreceptors can be found in the inner layer of the patient ROs. The retention of the rod photoreceptor might be the result of cGMP accumulation, which can be found at D193 and reaches a higher level at D230. Thus, we show that patient ROs recapitulate the late-onset disease phenotype at approximately D230 and resemble the impairments in photoreceptor maturation seen in RP. This patient-specific model could provide a promising platform for a disease model that is more advantageous than animal models.

## Materials and Methods

### Isolation and Expansion of Mononuclear Cells

To isolate mononuclear cells, 10 ml of peripheral blood was collected from a proband patient with a *PDE6B* mutation and an unaffected volunteer with no retinal disease and no mutation at any RP related genes following the instructions for Lymphoprep (Cat. #07851; Stemcell Technologies, Norway). Cells were maintained in StemSpan SFEM II medium (Cat. #09605; Stemcell Technologies, Canada) supplied with StemSpan Erythroid Expansion Supplement (Cat. #02692; Stemcell Technologies, Canada) for seven days for cell expansion. Both the patient and control volunteers were signed informed content, which has been approved by the Ethics Committee of the Eye Hospital of Wenzhou Medical University. The patient and control iPSCs were included in the hiPSC bank of Institute of Stem Cell Research, Wenzhou Medical University and designated as 502-PBMC-PDE6B-01 and 502-PBMC-HEALTHY-01.

### Generation and Characterization of Human iPSCs

Mononuclear cells were collected and subjected to a plasmid-based reprogramming system. To generate iPSCs, Episomal iPSC Reprogramming Plasmids (Cat. #SC900A-1; System Biosciences, United States) expressing four Yamanaka factors (Oct4, Sox2, Lin28, Klf4, and L-Myc), p53shRNA, and a miR-302/367 cluster were transformed into mononuclear cells using 4D Nucleofector (LONZA). iPSC colonies appeared after approximately 25 days. In this study, three independent colonies were picked for expansion and three colonies were used for the stepwise differentiation. Genomic DNA was extracted for amplification of the *PDE6B* gene and PCR products were subjected to Sanger sequencing.

Human iPSCs were maintained on Matrigel-coated (Cat. #356231; BD Corning, United States) dishes in mTeSR-E8 medium (Cat. #05940; Stemcell Technologies, Canada) and passaged with 0.5 μM EDTA (Cat. #AM9261; Ambion, United States). For trilineage differentiation (Deng et al., 2018), iPSC aggregates (5 to 10 cells) were cultured in suspension within DMEM/F12 medium (Cat. #11320033; Gibco, United States) supplied with 20% KSR (Cat. #10828028; Gibco, United States), 0.1 mM 2-mercaptoethanol (Cat. #M7522; Sigma, United States), 0.1 mM non-essential amino acids (Cat. #M7145; Sigma, United Kingdom), 2 mM GlutaMAX (Cat. #35050061; Life Technologies, Japan), 10 mM Y-27632 (Cat. #S1049; Selleckchem, United States), 100 U/ml penicillin, and 100 mg/ml streptomycin (Cat. #15140-122; Gibco, United States) for 8 days to form embryoid bodies. For spontaneous differentiation, the embryoid bodies were transferred into DMEM/F12 medium supplied with 10% FBS (Cat. #04-002-1A; Biological Industries), 0.1 mM 2-mercaptoethanol, 0.1 mM non-essential amino acids, 2 mM GlutaMAX 100 U/ml penicillin and 100 mg/ml streptomycin, and attached to glass slides coated with 0.1% gelatin (Cat. #ES-006-B; Millipore, Germany) for 10 days.

### Differentiation of 3D ROs

Three colonies, from the patient and control each were subjected to ROs generation. ROs were generated from iPSCs following a published method (Nakano et al., 2012) with slight modification. Briefly, iPSCs were dissociated into single cells with TrypLE Express (Cat. #12563-011; Gibco, Denmark) containing 0.05 mg/ml DNase I (Cat. #11284932001; Roche) and 20 μM Y-27632 resuspended in retinal differentiation medium I, and G-MEM medium (Cat. #11710-035; Gibco, United States) supplied with 20% KSR, 3 μM IWR1e (Cat. #681669; Merck Millipore, United States), 0.1 mM non-essential amino acids, 0.1 mM 2-mercaptoethanol, 1 mM pyruvate, 100 U/ml penicillin, and 100 mg/ml streptomycin. Approximately 12,000 cells in 100 μl were added to each well and reaggregated in a V-bottom low-cell-adhesion 96-well plate (Cat. #MS-9096V; Sumitomo Bakelite, Japan). The differentiation starting day was defined as day 0; 20 μM Y-27632 was added to the retinal differentiation medium I on day 0, and half the medium was exchanged with fresh retinal differentiation medium I on day 6. From day 2 to day 18, Matrigel was added in a final proportion of 1% v/v. On day 12, the cell aggregates were transferred to petri dishes in retinal differentiation medium II, G-MEM medium supplied with 10% (v/v) FBS, 100 nM SAG (Cat. #ALX-270-426-M001; Enzo Life Sciences, United States), 0.1 mM non-essential amino acids, 0.1 mM 2-mercaptoethanol, 1 mM pyruvate, 100 U/ml penicillin, and 100 mg/ml streptomycin. From day 18, each cell aggregate was cut into 3–5 small pieces and were maintained in neural retina culture medium, containing DMEM/F12-GlutaMAX medium (Cat. #10565-018; Gibco, United States) supplied with 10% (v/v) FBS, N2 supplement (Cat. #17502-048; Gibco, United States), 0.5 μM retinoic acid (Cat. #R2625; Sigma, United States), 12.5 μg/ml taurine (Cat. #T0625; Sigma, Japan), 100 U/ml penicillin and 100 mg/ml streptomycin. From days 18 to 30, about 50 organoids were maintained in a 100 mm Petri dish with 15–20 ml medium. After day 45, number of organoid in each dish was reduced to 30 with 15–20 ml medium. The neural retina culture medium was used from day 18 since after and changed weekly.

### Immunostaining and Imaging

Cells from trilineage differentiation were stained with markers representing the endoderm, mesoderm, and ectoderm. Cryosections of the ROs were stained with neural retina cell markers. Briefly, the cells were fixed in Immunol Staining Fix Solution (Cat. # P0098; Beyotime, China) for 10 min. Then, the cells or cryosections were permeabilized with 0.2% Triton X-100 (Cat. #A600198-0500; Sangon Biotech, China) for 10 min at room temperature. After rinsing twice with PBS, the cells or cryosections were incubated in blocking buffer (PBS containing 4% BSA) for 1 h to block non-specific antibody binding. Then, the cells or cryosections were incubated in blocking buffer with a primary antibody, including OCT4 (Cat. #ab18976; Abcam), SOX2 (Cat. #sc-17319; Santa Cruz), NANOG (Cat. #ab80892; Abcam), SSEA4 (Cat. #ab16287; Abcam), GFAP (Cat. #HPA056030; Sigma), α-SMA (Cat. #A5228; Sigma), AFP (Cat. #MAB1368; R&D System), PAX6 (Cat. #PRB-278P; Covance), CRX (Cat. #H00001406-M02; Abnova), NRL (Cat. #AF2945; R&D System), Recoverin (Cat. #AB5585; Millipore), Brn3b (Cat. #sc-514474; Santa Cruz), VSX2 (Cat. #sc-21690; Santa Cruz), RHO (Cat. #O4886; Sigma), M-opsin (Cat. #AB5405; Millipore), ARL13B (Cat. #17711-1-AP; Proteintech), PKCα (Cat. #P4334; Sigma), and Synaptophysin (Cat. #MA1-213; Invitrogen) for 12 h at 4°C. After being washed twice with PBS, the cells or cryosections were stained for 1 h with a fluorescence-conjugated secondary antibody, including Alexa Fluor 488 donkey anti-rabbit IgG (H + L) (Cat. #A-21206; Invitrogen), Alexa Fluor Plus 488 goat anti-mouse IgG (H + L) (Cat. #A32723; Invitrogen), and Alexa Fluor 594 donkey anti-mouse IgG (H + L) (Cat. #A21203; Invitrogen). After washing twice with PBS, cells or cryosections were stained for 10 min with DAPI (Cat. #GD3408; Genview). Images were recorded with an inverted confocal microscope (Leica SP8; Germany).

### Transcriptome Analysis and Quantitative PCR

For RNA-seq, total RNA from 3 to 5 organoids from two independent differentiations was isolated using TRIzol Reagent (Cat. #15596018; Invitrogen) and an RNeasy Plus Mini Kit (Cat. #74104; Qiagen) following the manufacturer’s instructions. RNA concentration was determined with a Nanodrop 2000 (ThermoFisher Scientific, United States). RNA-seq data was analyzed using BMKCloud^1^. Briefly, sequencing libraries were generated using NEBNext Ultra RNA Library Prep Kit for Illumina (#E7530L, NEB, United States) following the manufacturer’s recommendations and index codes were added to attribute sequences to each sample (Annoroad Gene Technology, China). The clustering of the index-coded samples was performed on a cBot Cluster Generation System using TruSeq PE Cluster Kit v4-cBot-HS (Illumia) according to the manufacturer’s instructions. Raw data (raw reads) of fastq format were firstly processed through in-house perl scripts. In this step, clean data (clean reads) were obtained by removing reads containing adapter, reads containing ploy-N and low quality reads from raw data. Hisat2 tools soft were used to map with reference genome.

For qPCR, total RNA from 6 to 10 organoids from two independent differentiations was isolated using TRIzol Reagent (Cat. #15596018; Invitrogen) following the manufacturer’s instructions. Total RNA was reverse-transcribed into cDNA using M-MLV Reverse Transcriptase (Promega; Cat. #M1705). The cDNA samples were used for quantitative PCR in a real-time PCR system (LightCycler 96 System; Roche, Mannheim, Germany) using a master mix (FastStart Universal SYBR Green Master [ROX]; Roche). Primer sequences are listed in Supplementary Table S1. The expression levels were normalized to the housekeeping gene glyceraldehyde-3-phosphate dehydrogenase (GAPDH) using the deltadelta Ct method.

### Enzyme-Linked Immunosorbent Assay (ELISA) of cGMP

cGMP levels in ROs were measured by ELISA using a cGMP direct immunoassay kit (Cat. #ab65356; Abcam). The ROs were homogenized in 0.1M HCl, and the supernatant was used for cGMP examination following the manufacturer’s instructions. The absorbance at 450 nm was measured using a SpectraMax M5 microplate reader (Molecular Devices). Each sample was prepared from three organoids that were selected with or cut into similar sizes. Each sample was detected in triplicate, and the results are an average of three or six independent experiments.

### Statistical Analysis

Quantitative analysis of each dataset was performed by two individuals who were blinded to the samples. For the analysis of rod and cone number, images of 1.29 mm × 1.29 mm in square and 15 μm confocal Z-stacks from three organoids (six images each for rod and four images for cone) of two independent differentiations from patient and control group each were acquired. Similarly, for cilia analysis, total images of 144.72 μm × 144.72 μm in square and 15 μm confocal Z-stacks from three organoids (three images each) of two independent differentiations were performed. Cilia were defined by ARL13B positive signals. Statistical analysis was performed with GraphPad Prism version 5.0 and SPSS 16.0. A two-tailed *t*-test or one-way ANOVA was used for comparison. A statistically significant difference was defined as *P* < 0.05.

## Results

### Generation and Characterization of Patient-Specific iPSCs Harboring a *PDE6B* Mutation

**FIGURE 1:**
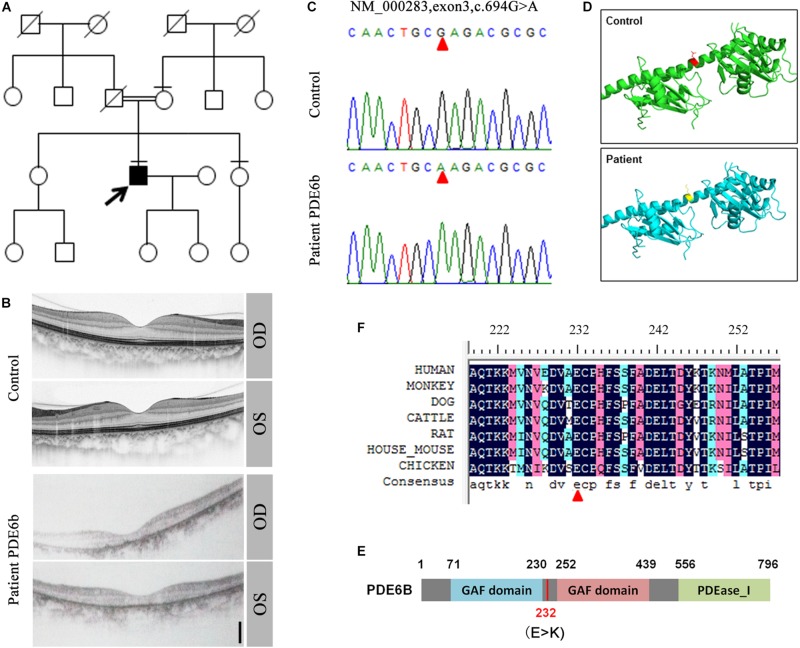
Identification of the PDE6B mutation in the RP patient. **(A)** The pedigree of the family. **(B)** Optical coherence tomography (OCT) of the RP patient and a healthy control. **(C)** Sequencing results of the RP patient and control. A single nucleotide mutation was found in the RP patient compared with the control and is indicated with red arrows. **(D)** Predicted crystal structures of the wild-type and mutant *PDE6B*. Amino acid 232 is indicated in red and yellow. **(E)** The domain organization of human PDE6B. The *PDE6B* c.694G > A mutation is located in the intervening sequence of two GAF domains arranged in tandem. **(F)** Multiple sequence alignment of PDE6B of the indicated species showing conserved amino acid residues at the mutation site which are indicated with a red arrow.

In a consanguineous family, a proband patient with night blindness was diagnosed with RP in his late 40s (Figure 1A). Severe loss of the photoreceptor layer was observed using optical coherence tomography (OCT) compared with the healthy contemporary control OCT (Figure 1B). To identify its genetic cause, targeted exon sequencing of 164 known retinal disease genes (Huang et al., 2015) was performed. A homozygous mutation within the *PDE6B* gene (c.694G > A) was identified, and direct Sanger sequencing validated the mutation (Figure 1C). This mutation was localized in the intervening sequence of two GAF domains arranged in tandem, causing the glutamate to change to lysine (232E-K) (Figures 1D,E). This amino acid is conserved across various species (Figure 1F).

**FIGURE 2:**
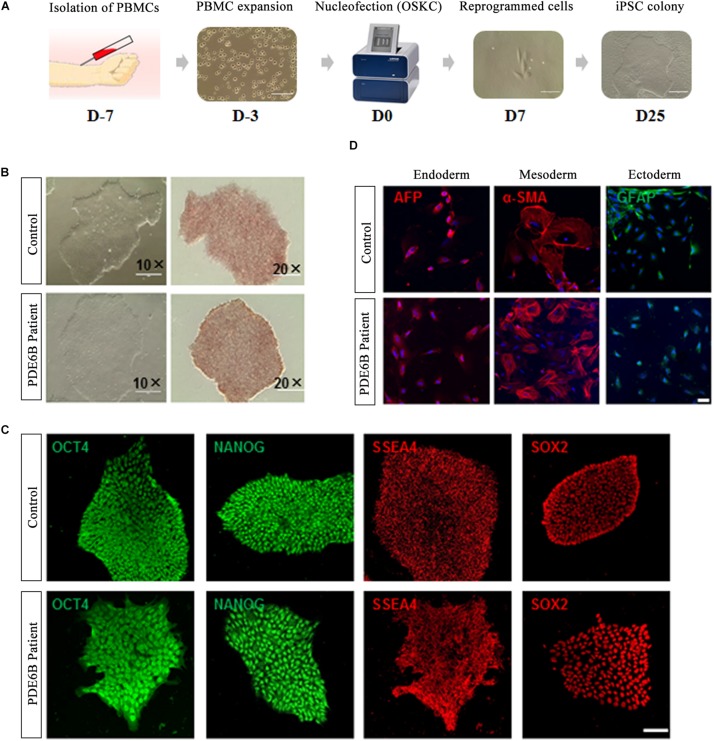
Generation and characterization of iPSCs of RP patient and non-phenotype control. **(A)** Timeline of iPSC generation. **(B)** Phase-contrast imaging (left; bar: 400 μm) and AP staining of iPSCs (right; bar: 200 μm). **(C)** Immunostaining of pluripotent markers. Bar: 50 μm. **(D)** Trilineage differentiation of iPSCs into three germ layers, endoderm (AFP^+^), mesoderm (α-SMA^+^) and ectoderm (GFAP^+^). Bar: 75 μm.

Peripheral blood mononuclear cells isolated from the PDE6B patient and an unaffected control individual were reprogrammed with a non-integrated method (Figure 2A). Three iPSC lines were derived from the patient and control and tested for AP staining and pluripotency markers (Figures 2B,C). Trilineage differentiation demonstrated that the iPSCs from the patient and control were capable of differentiation into three germ layers (Figure 2D). These results demonstrated the successful generation of patient-specific iPSCs.

### Generation of Long-Lived ROs for Disease Modeling

**FIGURE 3:**
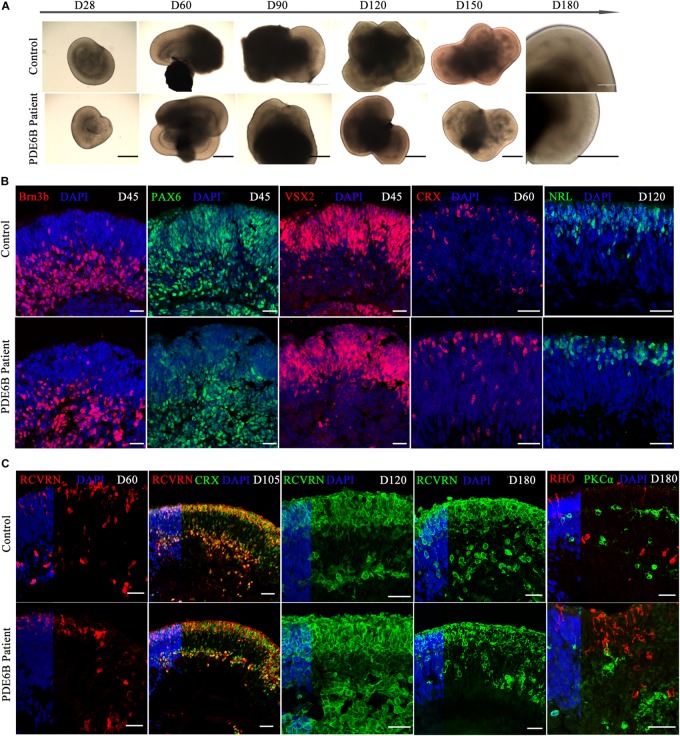
Comparison of retinal development between patient and control iPSC-derived organoids. **(A)** Phase-contrast images of retinal organoids from the patient and control at the indicated time points. Bar: 400 μm. **(B,C)** Immune fluorescence images of retinal organoids from the patient and control at the indicated time points. Brn3b, retinal ganglion cell marker; PAX6, retinal progenitor marker; VSX2, retinal progenitor marker; CRX, photoreceptor precursor marker; NRL, rod precursor marker; RCVRN, photoreceptor and cone bipolar marker; RHO, rod photoreceptor marker; PKCα, rod bipolar marker. Bar: 25 μm.

Human ROs were generated following the method described by Nakano et al. (2012) and Rossi et al. (2018). ROs derived from both patient and control iPSCs exhibited similar morphology and neural retina structure until D180 (Figure 3A). Structurally, the stratified architecture was clearly visible in phase-contrast imaging at D180 (Figure 3A). Brn3b expresses in retinal ganglion cells (RGCs) and VSX2 expresses in retinal progenitor cells (RPCs). PAX6 has a broad expression pattern, including RPCs, RGCs, amacrine and horizontal cells. Here, we chose Brn3b, PAX6 and VSX2 as RGC and RPC marker at the early-stage of retinal organoid. The expression patterns of Brn3b, PAX6 and VSX2 were similar in patient and control ROs at D45 (Figure 3B). By D45, neural retinal structures were formed and showed the expression of RGCs in the inner layer of the organoid.

CRX expresses in photoreceptor precursors and mature photoreceptors and NRL expresses in rod precursors and mature rods. At early-stage of ROs, CRX and NRL were identified as photoreceptor precursors and rod precursor marker. No obvious differences were found in CRX expression at D60 or in NRL expression at D120 (Figure 3B). The signal of the photoreceptor-specific marker CRX was spread through the inner and outer layers at D60 (Figure 3B) and aligned mainly at the outer layer at D105 (Figure 3C), indicating a stratified architecture similar to the early postnatal retina *in vivo* (Hendrickson et al., 2008). The expression of RCVRN appeared at approximately D60 and increased with the development of the ROs (Figure 3C). At D105, most of the CRX^+^ photoreceptor cells were coexpressed with RCVRN (Figure 3C), consistent with previous reports (Nakano et al., 2012). Immunostaining analysis showed that the ROs recapitulated a synchronized onset and progression of retinogenesis, and no obvious differences were observed in retinal progenitors or photoreceptor precursors between the patient and control ROs before D120. As RO development progressed, however, the expression of a rod photoreceptor marker (RHO) and rod bipolar cell marker (PKCα) showed obvious differences in patient ROs compared with control ROs at D180 (Figure 3C). Thus, patient ROs were successfully generated and showed obvious rod photoreceptor defects.

### Distinct Transcriptome of Late-Stage *PDE6B* Patient ROs

**FIGURE 4:**
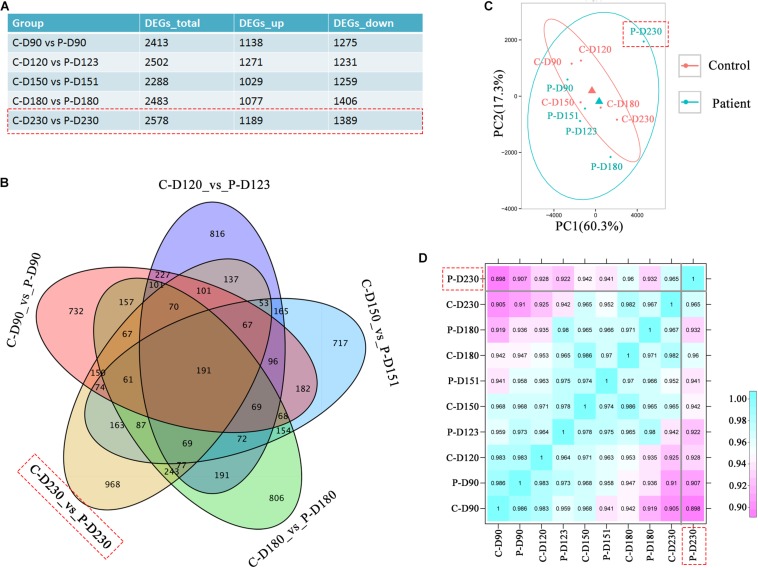
Transcriptome analysis identified defects in patient retinal organoids at D230. **(A)** Table of differentially expressed gene numbers in the control and patient organoids at the indicated time points. Total RNA of each sample was extracted of 3–5 organoids of two independent differentiations. **(B)** Venn diagram showing the number of genes differentially expressed in the control and patient and their overlaps at the indicated time points. **(C)** PCA of RNA-seq samples. **(D)** Correlation analysis of RNA-seq samples.

To investigate the transcriptional effects of the *PDE6B* c.694G > A mutation, comparison of bulk RNA-seq profiles were performed in patient and control ROs, which were collected from the mid-stage (D90, 120, 150, and 180) to late-stage (D230). A total of 2578 differentially expressed genes were found between patient and control ROs at D230 compared with the other tested time points (Figure 4A). Among these genes, 968 genes were not found at other time points (Figure 4B). Principal components analysis (PCA) of RNA-seq data indicated that the most variance was in PC1, which separated the patient ROs at D230 from the other samples (Figure 4C). This result was consistent with previous morphology and immunostaining results, indicating that a mild influence was found in mid-stage patient ROs. Furthermore, correlation analysis also confirmed the difference of patient ROs at D230 from the other ROs (Figure 4D). These results suggest that the *PDE6B* c.694G > A mutation may cause late-onset retinal disease, which is consistent with the latent RP clinical phenotype.

### Mislocalization of Rod Photoreceptors in Late-Stage ROs From the Patient

**FIGURE 5:**
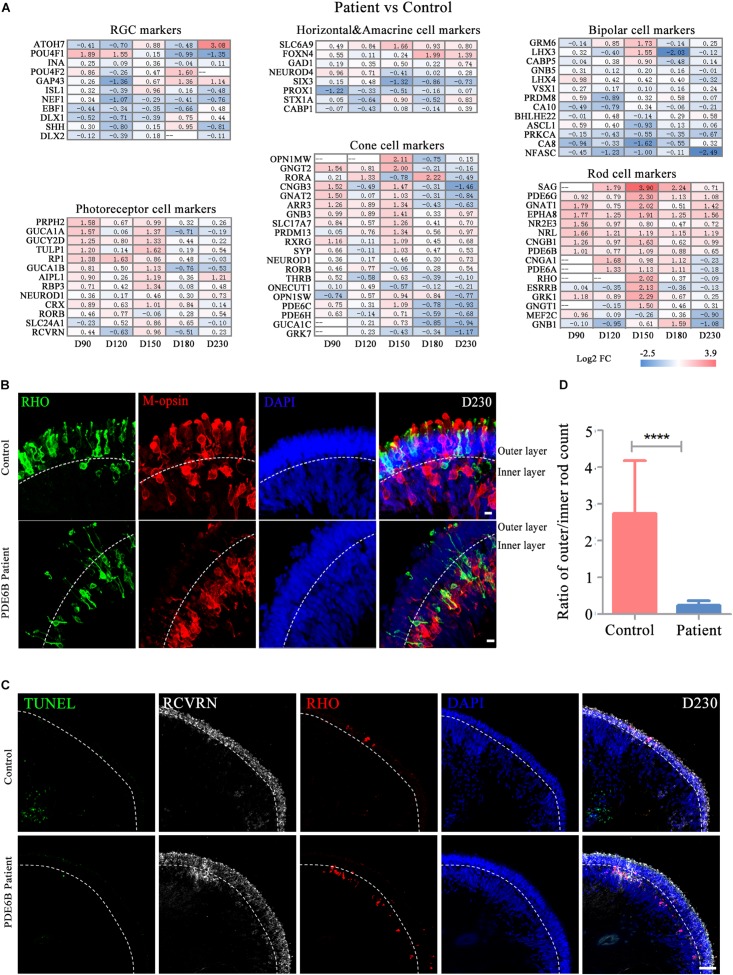
Defects in rod cell migration in patient retinal organoids at D230. **(A)** Changes in marker gene expression of retinal cell types were compared for control versus patient organoids. **(B)** Representative pictures of immunostaining analysis of rod and cone marker gene expression in control and patient organoids. Bar: 10 μm. **(C)** Representative pictures of TUNEL staining and immunostaining of RCVRN and RHO. Bar: 50 μm. **(D)** The ratio of rod numbers in the outer layer to the inner layer indicated in **(B)**. Statistical results are the mean values ± SD of 3 organoids (six images each) (D230) of two independent differentiations from the control and patient. Two-tailed *t-*test, ****P < 0.0001.

To further decipher the defects in late-stage *PDE6B* patient ROs, gene expression profiles of different retinal cell types, including RGCs, bipolar cells, horizontal and amacrine cells, photoreceptor cells, cone cells and rod cells, were compared between the patient and control ROs (Figure 5A). Among these retinal cell types, the expression of genes related to photoreceptor cell genesis, especially rod cell markers, was significantly higher in patient ROs than in control ROs (Figure 5A). Upregulation of transcription factors or regulators that promote rod development (SAG, NR2E3 and NRL) as well as genes related to phototransduction in rods were found at the mid-stage and decreased to levels relatively similar to those of the control at the late stage (Figure 5A). This abnormality in gene expression indicated rod development defects in the patient ROs.

Strong RHO expression was observed in both the control and patient ROs at D230. However, the distribution of the RHO signal was very different between the control and patient ROs (Supplementary Figure S1A). In the control ROs, RHO^+^ rod cells and M-opsin^+^ cone cells were mainly located in the outer layer with an outer-segment-like structure, indicating the mature morphology of photoreceptors. In contrast, most of the RHO and M-opsin signals appeared in the inner layer in patient ROs with immature morphology (Figure 5B). RCVRN positive cells and layered DAPI positive signal indicated outer layer clearly (Figure 5C). Few TUNEL positive cells were observed and no significant differences were found either at D180 (Figure 5C and Supplementary Figure S1B) or D230 (Figure 5C). Statistical analysis of three ROs (six images each) suggested that the ratio of rod cells in the outer and inner layers in the control ROs was significantly higher than that in the patient ROs (Figure 5D). The number of cones was also counted and standardized with ROs perimeter. However, no significantly differences were shown between control and mutation (Supplementary Figure S1C). Thus, these results implied that the *PDE6B* mutation complementarily deregulated rod cell marker expression in mid-stage ROs and specifically impaired rod cell maturation in late-stage ROs, and cell death might not be the main cause of rod mislocation.

### Functional Defects of *PDE6B* Patient ROs

**FIGURE 6:**
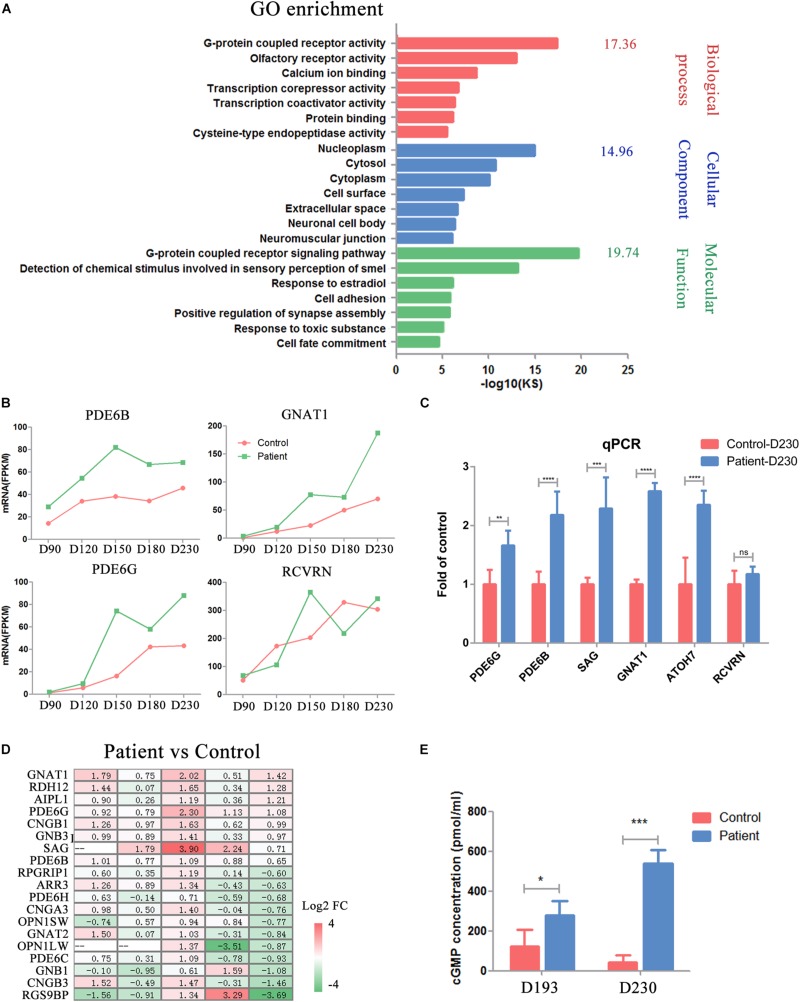
Impaired phototransduction was found in PDE6B patient retinal organoids. **(A)** GO analysis of differentially expressed genes for control and patient organoids at five different time points indicated in RNA-seq. **(B)** Expression of key genes in phototransduction (PDE6B, GNAT1, PDE6G) and photoreceptor and cone bipolar marker (RCVRN) at different time points included in the RNA-seq analysis. Total RNA of each sample was extracted of 3–5 organoids of two independent differentiations. **(C)** Representative qPCR results of two independent experiments. The results are the mean values ± SD of three replicates of each sample with 3–5 organoids. Two-tailed *t*-test, **P < 0.01, ***P < 0.001, ****P < 0.0001, ns *P* ¿ 0.05. **(D)** Changes in the expression of genes in the cGMP signaling pathway compared to patient versus control organoids. **(E)** Concentration of cGMP in retinal organoids. The results are the mean values ± SD of three independent experiments with three organoids in each sample of Control D193, Control D230 and Patient D230, and six independent experiments with three organoids in each sample of Patient D193. LSD test, *P < 0.5, ***P < 0.001.

PDE6B plays an important role in cGMP hydrolysis, which is a key part of phototransduction. Thus, we sought to gain further insights into the functional defects caused by the *PDE6B* mutation in ROs. Gene Ontology (GO) analysis of the transcripts significantly different at D230 between the patient and control ROs indicated an enrichment of genes implicated in G-protein-coupled receptor activity, G-protein-coupled receptor signaling pathway and calcium ion binding (Figure 6A). Higher expression of G-protein subunit alpha transducing 1 (GNAT1), a stimulator of rhodopsin and cGMP-phosphodiesterase coupling, PDE6B and PDE6G, and the beta and gamma subunits of cyclic GMP-phosphodiesterase, which are key players in stimulating cGMP hydrolysis in rods during visual impulses, was identified in patient ROs at D230 (Figure 6B). To validate the RNA-seq results, qPCR was performed with several target genes including SAG, PDE6B, GNAT1, PDE6G, RCVRN, and ATOH7 (Figure 6C). The expression of PDE6G, PDE6B, GNAT1, SAG, and ATOH7 were significantly higher in the patient ROs than that in the control. The level of RCVRN expression was similar in the patient and control ROs at D230. These results were consistent with the RNA-seq. Accompanied by slightly different RCVRN expression, indicating a similar number of cells in the patient and control ROs, these extraordinary changes implied impaired cGMP hydrolysis in the patient ROs at D230 (Figure 6B). A prominent change in the expression of genes involved in the cGMP-PKG signaling pathway was observed, suggesting abnormal cGMP levels in the patient ROs at D230 (Figure 6D). Genes that promote cGMP hydrolysis, such as the essential component for rod cGMP-phosphodiesterase biosynthesis (AIPL1), were significantly deregulated in ROs at D230 (fold change > 2) (Figure 6D). Conversely, genes involved in the G-protein-coupled receptor signaling pathway and ion channel, such as G-protein subunit beta 1 (GNB1), regulator of G-protein signaling 9 binding protein beta, and subunit of a cyclic nucleotide-gated ion channel (CNGB3), were profoundly downregulated in ROs at D230 (fold change > 2) (Figure 6D). These results implied that the cGMP level in the patient ROs might be affected.

Thus, the concentrations of cGMP in the ROs were detected by ELISA at D193 and 230. At D193, a significantly higher cGMP concentration was found in patient ROs. As the ROs developed into the late stage (D230), this difference was remarkably increased, reaching almost 10 times higher in patient ROs than in control ROs (Figure 6E). These results suggested that the *PDE6B* mutation impaired its function in cGMP hydrolysis, leading to increased cGMP levels in ROs.

**FIGURE 7:**
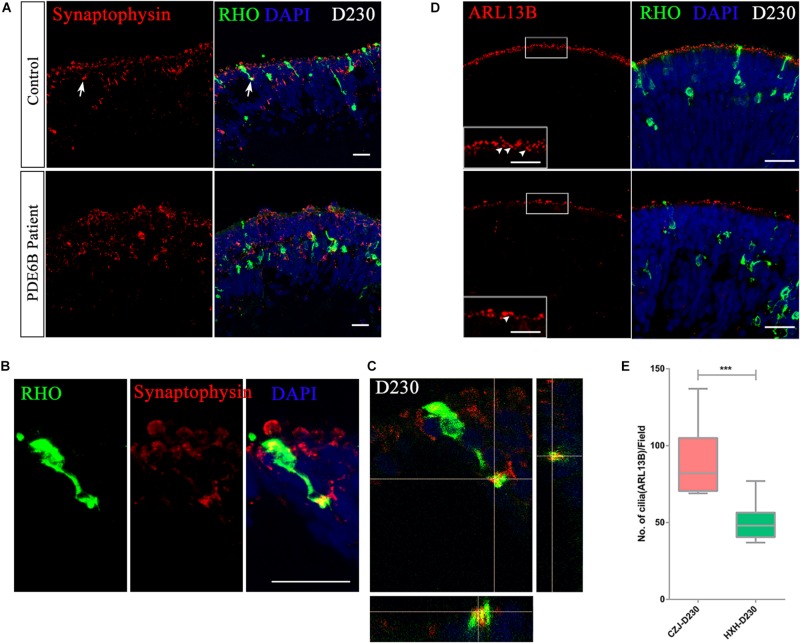
Impaired rod maturation in PDE6B patient retinal organoids. **(A)** Representative immunostaining pictures of the presynaptic marker synaptophysin and rhodopsin (RHO) in retinal organoids. Bar: 25 μm. **(B)** Amplified presynaptic formation of rod photoreceptors indicated with arrows in **(A)**. Bar: 25 μm. **(C)** Colocalization of RHO and synaptophysin. **(D)** Representative immunostaining pictures of the cilium marker ARL13B and RHO in retinal organoids at D230. Bar: 25 μm. Boxes were amplified images. White arrows indicated definite signal of ARL13B represent cilia. Bar: 6.25 μm. **(E)** Statistic analysis of cilia number in retinal organoids at D230. Statistical results are the mean values ± SD of 3 organoids (3 images each) (D230) of two independent differentiations from the control and patient. Two-tailed *t*-test, ***P < 0.001.

To further identify whether the accumulated cGMP affects the formation of synaptic connections, the expression of the synaptic vesicle membrane protein, synaptophysin, was analyzed at D230. A distinct expression pattern of synaptophysin was observed in the control and patient ROs (Figure 7A). In the control ROs, most of the synaptophysin signal formed condensed vesicles located in the two apical regions of the outer nuclear layer. However, synaptophysin was spread through the cytoplasm in the patient ROs (Figure 7A). In addition, the presynaptic structure was formed at the end of the rod photoreceptor cells in the control ROs (Figures 7B,C and Supplementary Movie). No similar structure was found in the patient ROs.

The connecting cilium is an important structure for material transfer between the outer segment and inner segment in photoreceptor cells. The formation of the connecting cilium can be considered a hallmark of photoreceptor cell maturation. Thus, we also examined the connecting cilium via ARL13B expression in ROs at D230 (Figure 7D and Supplementary Figure S1D). A remarkably reduced number of cilia was observed in *PDE6B* patient ROs (Figure 7E), which is more obvious in 3D reconstruction images (Supplementary Figure S1D). Overall, in the *PDE6B* mutant RO model, elevated cGMP levels are suspected to be linked to impaired rod migration from the inner layer to the outer layer and impeded formation of synapses and the connecting cilium in photoreceptor cells.

## Discussion

The heterogeneity of RP inevitably promotes the difficulty of the development of treatment or therapeutic methods. Although animal models have provided invaluable tools for mechanism unveiling and drug testing; large gaps in understanding gene variability and structural discrepancy remain. With the development of new technologies comes the need to obtain more precise models of the human retina to study this highly organized tissue. With the development of 3D culture of stem cell-derived ROs *in vitro*, researchers can now generate well-structured retina-like tissue and mature photoreceptors possessing the appropriate electrophysiological properties and photosensitivity. Transcriptome analysis has demonstrated that ROs are excellent resources for studying retinal development (Kaewkhaw et al., 2015; Voelkner et al., 2016). Eldred and colleagues have used hESC-derived ROs as a model for determining the mechanism that controls photoreceptor cell fate during human retinal development (Eldred et al., 2018). Combined with single-cell sequencing of cells isolated from hESC-derived ROs, critical pathways and novel genes regulating retinal progenitor cell commitment have been found (Mao et al., 2019).

Using patient-derived stem cells, patient-specific ROs can be established to better understand pathogenesis and to test new therapeutics. To compensate for the inconsistency of ROs derived from different iPSC lines with varied methodologies, Capowski et al. (2019) promoted a staging system for RO differentiation. Here, we have analyzed the disease phenotype and expression profile within a staging system, which is largely consistent with their findings. Based on the staging system, the RO disease models established to date have all been early- or mid-stage (Parfitt David et al., 2016; Shimada et al., 2017; Teotia et al., 2017; Buskin et al., 2018; Deng et al., 2018; Guo et al., 2019; Huang et al., 2019; Li et al., 2019; Quinn et al., 2019). For example, in a previous RPGR-RP model established in our lab (Deng et al., 2018), impaired gene expression was found as early as D90, and impaired photoreceptor morphology was observed at approximately D150. In addition, another PRPF31-RP11 model established by Buskin et al. (2018), impaired pre-mRNA splicing and impaired photoreceptor morphology was found at week 21 of differentiation. Notably, in the PDE6B RO model, a severe disease phenotype is observed only at D230, which is considered late-stage.

By now, 65 genes have been identified as disease-causing genes of RP^2^. Mutations in *PDE6B* cause recessive RP and dominant congenital stationary night blindness with vastly variable phenotypes. Here, we used a patient-derived RO model to demonstrate how the *PDE6B* mutation causes retinal degeneration in RP. Akin to the *rd1* and *rd10* mouse models (Bowes et al., 1990; Barhoum et al., 2008; Wang et al., 2018), this defect in PDE6B leads to an accumulation of cGMP in ROs at D193, and the accumulation increases to 10 times higher than that of control ROs as the ROs grow to D230 (Figure 6D). Additionally, defects in photoreceptors can be observed in the patient ROs, including mislocation and abnormal morphology at D230 (Figure 5), which is consistent with the photoreceptor death found in the mouse models at approximately P8 in *rd1* and P18 in *rd10* (Bowes et al., 1990; Gargini et al., 2007; Barhoum et al., 2008). Furthermore, the expression of cGMP metabolic-related genes was significantly changed in patient ROs at D230, indicating impaired photoreceptor function (Figure 7A). In summary, we have established a PDE6B-RP model with patient iPSCs exhibiting cardinal characteristics as predicted based on the phenotypes shown in animal models. Additionally, our findings have been drawn from three colonies of one patient and one healthy control. In future study, we will screen for more PDE6B-RP cases and perform gene correction as we reported previously (Deng et al., 2018).

Our hope is that the establishment of a late-stage RP model *in vitro* may provide a reasonable platform that will be utilized in drug screening in addition to rodent models. With a recent study demonstrating the successful infection of retinal organoids with AAV (Quinn et al., 2019) and rescue of CEP290 function with oligonucleotides in patient ROs (Dulla et al., 2018), this well-characterized patient-based system is promising for deciphering disease mechanisms, evaluating the efficacy of new drugs, and testing the efficiency of gene therapy before clinical trials.

## Data Availability Statement

The datasets generated for this study can be found in the Gene Expression Omnibus with accession number GEO: GSE141531.

## Significance Statement

Retinitis pigmentosa (RP) is a hereditary retinal degenerative disease, and 65 disease-causing genes have been identified. Animal models have been used for pathogenesis interpretation and drug testing, but conflicting results have been found because of interspecies variation. However, human iPSCs and a retinal organoid (RO) differentiation system provide an unlimited cell source for disease modeling and drug screening. Here, we developed patient ROs with a *PDE6B* mutation, and an obvious disease phenotype was found at differentiation day 230. Moreover, elevated cGMP levels and mislocalization of rod cells were observed. This patient-based late-onset RP model can be utilized to decipher the mechanisms underlying RP and evaluate new treatments.

## Ethics Statement

The studies involving human participants were reviewed and approved by The Eye Hospital of Wenzhou Medical University Ethics Committee. The patients/participants provided their written informed consent to participate in this study.

## Author Contributions

M-LG conducted the data analysis and interpretation, manuscript writing, financial support, and the final approval of the manuscript. X-LL carried out the collection and assembly of data, data analysis and interpretation, and the final approval of the manuscript. FH carried out the collection and assembly of data, data analysis, manuscript revision, and final approval of manuscript. KW-H carried out the collection and assembly of data. S-QJ carried out the assembly of the data. Y-YZ carried out the data collection. Z-BJ conceptualized the design, provided the materials and the financial support for the study, and gave final approval for the manuscript.

## Conflict of Interest

The authors declare that the research was conducted in the absence of any commercial or financial relationships that could be construed as a potential conflict of interest.

## Footnotes

1 www.biocloud.net

2 https://sph.uth.edu/retnet
