# Supplementary figures and images for: Patient-Specific Retinal Organoids Recapitulate Disease Features of Late-Onset Retinitis Pigmentosa
Source: Front Cell Dev Biol. 2020 Mar 6;8:128. doi: 10.3389/fcell.2020.00128 (PMC7068133; doi:10.3389/fcell.2020.00128)

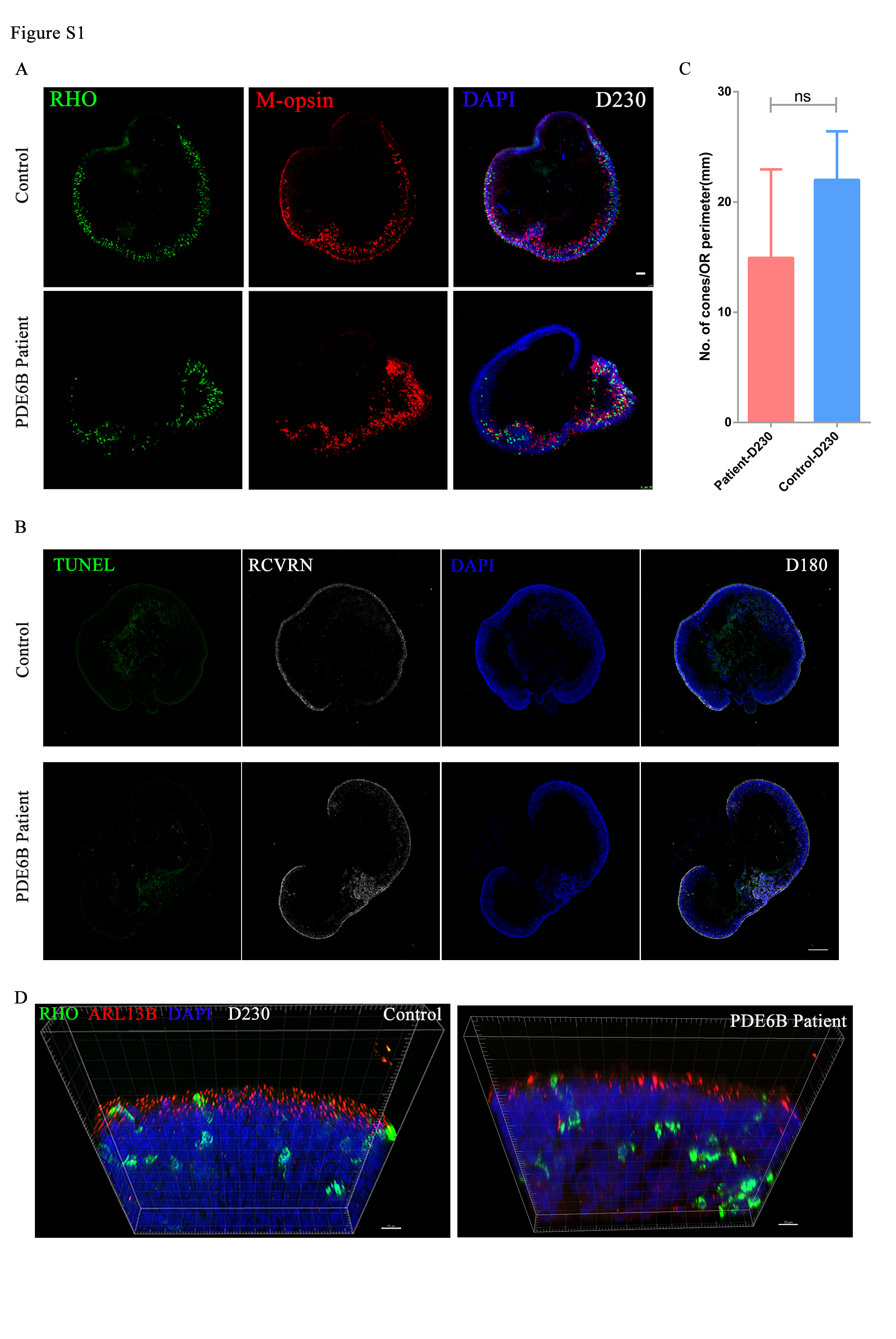

Supplement: Supplementary file 1 [file Image_1.TIF]
