# Supplementary material for: Patient-Specific Retinal Organoids Recapitulate Disease Features of Late-Onset Retinitis Pigmentosa
Source: Front Cell Dev Biol. 2020 Mar 6;8:128. doi: 10.3389/fcell.2020.00128 (PMC7068133; doi:10.3389/fcell.2020.00128)
Supplement: Supplementary file 2 [file Table_1.docx]

Table S1 Primer sets used for PCR and qPCR

| **Gene name** | **Forward** | **Reverse** | **Length of PCR products/bp** | **Application** |
| --- | --- | --- | --- | --- |
| PDE6B-mut | CTCAGGCGAGCATGTTTCT | TACCCAGGTGAGCACAAGA | 293bp | PCR |
| SAG | AAGACCAGCAAGTCCGAACC | GGATCAACCAACACGACACCA | 143bp | qPCR |
| PDE6B | GACGTGTGGTCTGTGCTGAT | CTTGCCGTGGAGGATGTAGTC | 111bp | qPCR |
| GNAT1 | GCCTCGGAGTACCAGCTCA | GCCAGTGGTCTTGACTCGC | 123bp | qPCR |
| RCVRN | TTCCAGACGATGAAAACACGC | GCCAGTGTCCCCTCAATGAA | 112bp | qPCR |
| ATOH7 | CTGCCTTCGACCGCTTACG | CAGAGCCATGATGTAGCTCAG | 104bp | qPCR |
| PDE6G | GACCAGGCAGTTCAAGAGCA | GTGGTTGAAGGCCTCCCAAG | 124bp | qPCR |
| GAPDH | GTCTCCTCTGACTTCAACAGCG | ACCACCCTGTTGCTGTAGCCAA | 131bp | qPCR |
